# Adsorption and Diffusion of Hydrogen in Carbon Honeycomb

**DOI:** 10.3390/nano10020344

**Published:** 2020-02-18

**Authors:** Qin Qin, Tingwei Sun, Hanxiao Wang, Pascal Brault, Haojie An, Lu Xie, Qing Peng

**Affiliations:** 1 School of Mechanical Engineering, University of Science and Technology Beijing, Beijing 100083, China; qinqin@me.ustb.edu.cn (Q.Q.); s20180483@xs.ustb.edu.cn (T.S.); anhaojie@xs.ustb.edu.cn (H.A.); 2 Reactor Engineering and Safety Research Center, China Nuclear Power Technology Research Institute Co., Ltd., Shenzhen 518031, China; wanghanxiao@cgnpc.com.cn; 3 GREMI UMR7344 CNRS, Université d’Orléans, BP6744, 45067 Orleans CEDEX 2, France; 4 Physics Department, King Fahd University of Petroleum & Minerals, Dhahran 31261, Saudi Arabia

**Keywords:** hydrogen adsorption, carbon honeycomb, molecular dynamics, grand canonical Monte Carlo simulations, pressure, temperature

## Abstract

Carbon honeycomb has a nanoporous structure with good mechanical properties including strength. Here we investigate the adsorption and diffusion of hydrogen in carbon honeycomb via grand canonical Monte Carlo simulations and molecular dynamics simulations including strength. Based on the adsorption simulations, molecular dynamics simulations are employed to study the effect of pressure and temperature for the adsorption and diffusion of hydrogen. To study the effect of pressure, we select the 0.1, 1, 5, 10, 15, and 20 bars. Meanwhile, we have studied the hydrogen storage capacities of the carbon honeycomb at 77 K, 153 K, 193 K, 253 K and 298 K. A high hydrogen adsorption of 4.36 wt.% is achieved at 77 K and 20 bars. The excellent mechanical properties of carbon honeycomb and its unique three-dimensional honeycomb microporous structure provide a strong guarantee for its application in practical engineering fields.

## 1. Introduction

Hydrogen is a renewable energy source that can be used to replace fossil fuels with promising application prospects. The key to utilize this clean energy source is the efficient storage of hydrogen [1]. An effective way to improve hydrogen storage is to increase external pressure. However, the hydrogen storage capacity rises quite slowly when external pressure reaches a certain value. The introduction of porous materials could be a way to solve these problems, which adsorb hydrogen with relatively low pressure [2]. Many research works show that porous materials including zeolites [3], carbon-based nanomaterials (CBNs), and metal organic frameworks (MOFs) [4] have certain hydrogen storage potential [5]. The design of porous materials with low density and high porosity plays an important role in hydrogen storage.

Adsorption storage in porous materials is a new way to store hydrogen, which is considered to be safe and reliable, and has high hydrogen storage efficiency [6]. Meanwhile, physisorption of hydrogen on the porous material surface requires relatively small external pressure, low cost and simple material structural design [2]. Nanoporous materials are considered to have the greatest potential in adsorption storage due to the high adsorption surface area. Ma et al. [7] prepared a porous structure and found that the porous structure has excellent adsorption characteristics. Among those known nanoporous materials, CBNs have lower density than both zeolites and MOFs [8]. It is reported that such materials uptake and release more easily [9]. In addition, CBNs show tunable porosity and surface area since there are many structural forms including graphene, fullerene, carbon nanotubes and their various combinations [10,11]. Therefore, it is worth exploring the potential of new kinds of CBNs in hydrogen adsorption storage.

There are lots of studies about the hydrogen adsorption inside porous carbon nanomaterials. Active carbon is known as a kind of carbon material with high specific surface area. The reported amount of adsorbed hydrogen is up to about 5 wt.% at 77 K, while it is about one order of magnitude lower at room temperature under the same pressure [12,13]. Wu et al. [14] reported that the amount of hydrogen physisorption in four-layer graphene sheets with interlayer spacing of 1.4 nm can reach 10 wt.%. Carbon nanotubes (CNTs) are also reported to have high hydrogen adsorption capacity [15,16]. Research shows that hydrogen adsorption on one side of graphene sheet can be up to 3 wt.% [17], and pillared graphene reported by Wu et al. can reach a hydrogen adsorption of 4 wt.% [11].

Although some of those various combinations of CNT, graphene and fullerene exhibit outstanding hydrogen storage capacity, synthesis is still challenging [18]. Recently the introduction of a template carbonization process provides a new method for the synthesis of carbon nanoporous materials, in which the template is carbonized and then removed to obtain a variety of carbon porous materials [19]. Khanin et al. [20] synthesised a carbon replica of zeolite experimentally. Thomas et al. [21] obtained some carbon nanoporous structures by carbonization of faujasite zeolite template, and investigated the adsorption capacity of these structures. In addition, a theoretical method has been performed to simulate and predict carbon replica of template. Joshua et al. [22] investigated the adsorption and diffusion of gas in a carbon replica of zeolite faujasite using the Grand Canonical Monte Carlo (GCMC) method. Efrem et al. [19] investigated the synthesis of zeolite-templated carbon (ZTC) using chemical vapour deposition technology and introduced a theoretical method to obtain the atomic structure of ZTC from any template. This makes it possible to successfully predict a carbon replica of any specific template, and gives carbon materials a more fascinating prospect in hydrogen storage.

Similar to ZTC, carbon honeycomb (CHC) has a nanoporous structure. Our previous study illustrates that CHC possesses outstanding mechanical properties [23], which could form a strong framework for hydrogen storage. It is natural to question to what extent the CHC can store hydrogen, which motivated us to carry out this investigation.

This study aims to explore the capability of the hydrogen storage in CHC to extend the horizon of the applications of CHC in hydrogen energy. It can be concluded from the literature [24] that during the adsorption process, quantum confinement induces disorder on the positional, orientational, and intramolecular structures of the adsorbed atoms. Therefore, the position, orientation and structure of hydrogen atoms are not our focus. The ability of CHC to store hydrogen is quantified by changes in the number of hydrogen atoms. The pressure and temperature effects on the hydrogen storage are examined while the carbon framework of CHC was kept fixed. GCMC simulations are employed to investigate the hydrogen storage capacity of CHC which has been experimentally synthesized recently [23,25,26]. Based on the model after hydrogen adsorption, molecular dynamics (MD) methods are utilized to investigate the diffusion of hydrogen confined in the carbon honeycomb. The effect of pressure and temperature are investigated. To study the effect of pressure, we select the 0.1, 1, 5, 10, 15 and 20 bars. In addition, we study the hydrogen storage capacities of the carbon honeycomb at 77 K, 153 K, 193 K, 253 K and 298 K.

## 2. Methodology

**Figure 1:**
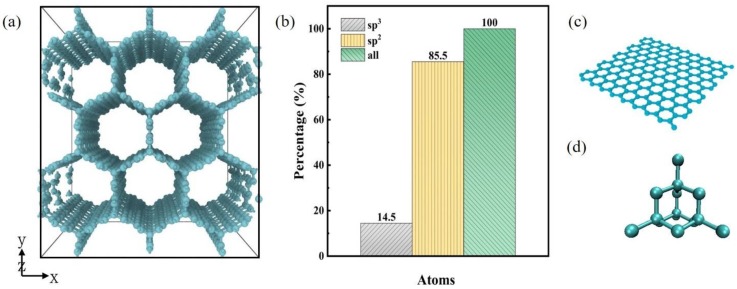
(**a**) The schematic diagraph of carbon honeycomb (CHC) atomic structures. (**b**) The percentage of hybrid method of carbon atoms. (**c**) The schematic diagraph of graphene. (**d**) The schematic diagraph of diamond. The hybridization of CHC at the junction atoms are sp^3^, and the remaining atoms are sp^2^. Graphene is a typical structure with sp^2^ hybridization of carbon atoms. Diamond is a typical structure with sp^3^ hybridization of carbon atoms.

The atomic structures of CHC and the percentage of carbon atoms’ hybrid method are presented in Figure 1. CHC has a high structural stability due to its sp^2^–sp^3^ hybridization and the sp^2^ and sp^3^ carbon atoms result in porous structures [27]. The hybridization at the junction atoms of CHC are sp^3^, and the remaining atoms are sp^2^. Graphene, a two-dimensional carbon structure, is a typical structure with sp^2^ hybridization of carbon atoms. It exhibits outstanding physical, chemical, mechanical properties, thermodynamic and other properties [28,29]. Diamond is a typical structure with sp^3^ hybridization of carbon atoms. Diamond is the hardest mineral in nature. It is widely used in precision grinding and other industries.

The density of CHC is 1.31 g/cm^3^. Hydrogen adsorption is related to the porosity of porous materials. Here, the Zeo++ package, an open source software, is employed to investigated the void space representations of carbon materials [30]. For each calculation, the number of Monte Carlo samples per atom is set to 100,000. The pore size distribution (PSD) is calculated with a small spherical probe of 0.5 Å radius so that more details can be detected. The pore size of CHC is 8.3 Å. It was reported that carbon materials with pore sizes between 7 and 12 Å have the most promising potential for hydrogen storage, because the transport efficiency is low when the pore is too small and the adsorption amount at room temperature is low when the pore is too large [17]. Therefore, the material used in this work is suitable for hydrogen adsorption. When calculating the accessible surface area (ASA) and accessible volume (AV) fraction, the spherical probe with radius of 1.625 Å is utilized [31,32]. The accessible surface area (ASA) and accessible volume (AV) fraction of CHC are 1071 m^2^/g and 12.32%, respectively.

Before the GCMC simulation, the CHC was relaxed by using isothermal-isobaric (NPT) ensemble to reach equilibrium via MD simulations. The external pressure was zero at the relax stage. The temperature in the relax stage was the same as that in the GCMC simulated, which are 77 K, 153 K, 193 K, 253 K and 298 K, respectively. The MD timestep was 1 fs and duration was 20 ps. Periodic boundary conditions along *x*, *y* and *z* direction were used.

After equilibrating the system at the corresponding temperature, the GCMC simulations were carried out to simulate hydrogen adsorption isotherms at 77 K, 153 K, 193 K, 253 K and 298 K by using the Large-scale Atomic Molecular Massively Parallel Simulator (LAMMPS) package (lammps-7 August 2019, Sandia National Laboratories, Albuquerque, USA) [33,34]. The Open Visualization Tool (OVITO) [35,36] package (3.0.0-dev646, the OVITO software, Darmstadt, GER) was used for structure and data analysis. In GCMC simulations the adsorption isotherms of hydrogen are calculated by balancing the chemical potential μ with an imaginary ideal gas zone at specific temperature T and pressure P, which is defined as [37]:

(1) $$\mu =k_{B}T\ln\frac{\phi P\Lambda^{3}}{k_{B}T}$$

where $k_{B}$ and Λ represent Boltzmann’s constant and thermal de Broglie wavelength, respectively. ϕ is the fugacity coefficient. adaptive intermolecular reactive empirical bond order (AIREBO) potential [38] is utilized to describe the interactions of carbon–carbon, carbon–hydrogen and hydrogen–hydrogen. This potential ensures that hydrogen exists in molecular form, which is in line with reality. The carbon structures are considered to be rigid in GCMC simulations. That is reasonable due to the high stiffness of those carbon structures and it has been used in many previous studies to improve the computational efficiency [10,11,39]. The number of grand canonical Monte Carlo (GCMC) steps for hydrogen adsorption is 200,000 in GCMC simulation. The boundary of the simulation boxes is considered as periodic and a time step of 0.1 fs is utilized. The timestep size is set for subsequent molecular dynamics simulations. The timestep units is associated with each choice of units that LAMMPS supports.

## 3. Results and Discussion

### 3.1. Mechanical Properties of Carbon Honeycomb

Materials used for hydrogen storage should not only have a high adsorption capacity, but also sufficient mechanical strength. In our previous research, it can be concluded that CHC has fascinating mechanical properties and potential application prospects [25,26]. We studied the mechanical properties of CHC when stretching along the different tilt angle in the zigzag–armchair (*x*–*y*) plane. It can be concluded that the mechanical properties of CHC in the *x*–*y* plane show anisotropy and the strength of CHC decreases with the tilt angle increasing, which is similar to that of graphene. The effect of temperature and vacancy-type defects on the mechanical properties of CHC were studied. The results show that temperature affects the strength of CHC and the strength of CHC decreases as the temperature increases. Vacancy defects affect the strength and fracture strain of the CHC. The strength is sensitive to the location and bonding of the vacancies.

CHC has great application potential in many aspects for the outstanding mechanical properties, which could be conducive to the storage of hydrogen. Hydrogen enters the internal pores of the CHC and exerts a force on the structure.

### 3.2. Pressure Effect on the Hydrogen Adsorption

**Figure 2:**
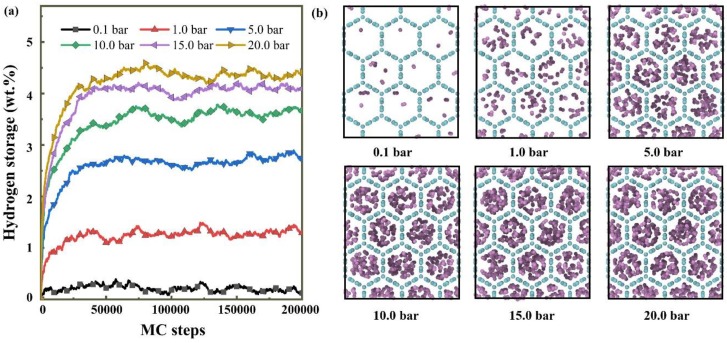
(**a**) The relationship between the mass fraction of adsorbed hydrogen and grand canonical Monte Carlo (GCMC) steps (**b**) the topography of 150,000 MC steps under different pressures at the temperature of 77 K for CHC.

The effect of pressure on the amount of hydrogen molecules adsorbed inside CHC materials is investigated at the temperature of 77 K. Figure 2 exhibits the variation of the mass fraction of adsorbed hydrogen and the number of MC steps at pressures ranges from 0.1 to 20 bar. It can be seen from Figure 2a that the hydrogen adsorption process in CHC could be divided into three stages: rapid adsorption, slow adsorption and saturation. The adsorption of hydrogen molecules on the surface of CHC occurs mainly at the relatively early stage of the GCMC simulations. The topography of different pressures is shown in Figure 2b during the saturation phase (at the MC steps of 150,000).

At the pressure of 0.1 bar, the amount of adsorbed hydrogen molecules reaches saturation rapidly, and this rapid adsorption stage lasts longer as the pressure rises. The chemical potential of hydrogen in the simulation box is then close to the given value. Therefore, the adsorption rate of hydrogen is gradually reduced until a stable saturation state is reached, indicating that the simulated hydrogen reservoir is balanced with the imaginary ideal hydrogen zone at specified chemical potential. The hydrogen adsorption processes from GCMC simulations are similar with previous report from MD simulation [11].

The amount of adsorbed hydrogen increases as the pressure increases, as shown in Figure 2. When the pressure is improved from 0.1 to 20 bar it increases 12-fold for CHC. The hydrogen adsorption is about 4.36 wt.% for CHC at the pressure of 20 bar. The hydrogen adsorption is about 0.3 wt.% for a single-walled carbon nanotube (SWNT) and 1.2 wt.% for SWNT after sonication in dimethyl formamide [40] at 80 K and 10 bar hydrogen pressure. It is 2.2 wt.% and 3.6 wt.% reported by Farida et al. for CNTs arranged in square and hexagonal lattices at 77 K and 10 bar [41]. The highest hydrogen adsorption for graphene oxide framework materials is around 1.2 wt.% at 77 K [42,43].

### 3.3. Temperature Effect on the Hydrogen Adsorption

To investigate the hydrogen adsorption isotherms of CHC, the simulation system was kept at a pressure range from 1 to 20 bar. Besides, temperature is another influencing factor affecting the hydrogen storage capacity of porous materials. Different temperature conditions (77 K, 153 K, 193 K, 253 K and 298 K) are utilized to analyze the effect of temperature on the hydrogen adsorption in the carbon structures.

**Figure 3:**
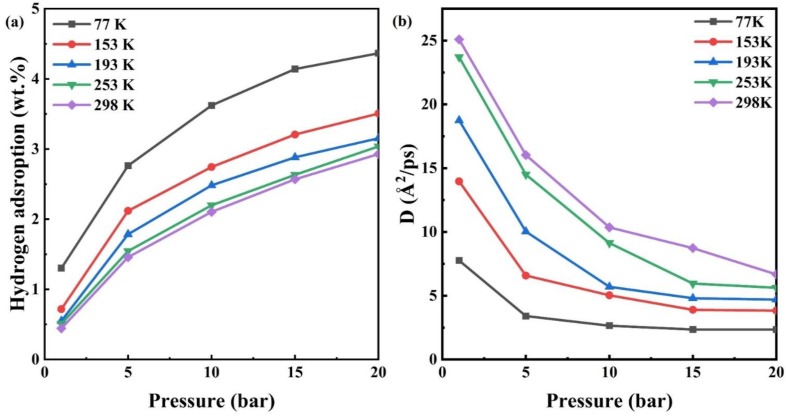
(**a**) The hydrogen adsorption isotherms and (**b**) the diffusion coefficients for hydrogen for CHC under different hydrogen pressure at the temperature of 77 K, 153 K, 193 K, 253 K and 298 K, respectively.

The hydrogen adsorption isotherms for CHC at 77 K, 153 K, 193 K, 253 K and 298 K are presented in Figure 3a. The isotherms are observed from Figure 3a, which corresponds to a previous report [10] since the carbon materials are microporous (pore size is less than 2 nm) structures. At the low-pressure region (<5 bar), the amount of hydrogen adsorption increases rapidly with increasing pressure, while the amount of adsorption tends to increase slowly at the high-pressure region (>10 bar). The maximum adsorption of hydrogen for CHC is about 4.36 wt.% at the temperature of 77 K and pressure of 20 bar. Langmi et al. [44] reported that the hydrogen adsorption in ion-exchanged zeolites is 2.19 wt.% for CaX, 1.96 wt.% for KX at 77 K and 15 bar. In addition, lots of research works show that the gravimetric storage capacity of zeolites is generally below 3 wt.% [2,3,45,46,47]. The result shows that CHC are more suitable for hydrogen storage than zeolites.

Figure 3a demonstrates that the hydrogen adsorption capacity of the CHC decreases as the temperature increases from 77 K to 298 K. The kinetic energy of hydrogen molecules increases with increasing temperature, so that more adsorption potential is required to adsorb hydrogen molecules with higher kinetic energy. Therefore, the increase in temperature is not conducive to the adsorption of hydrogen on the carbon nanoporous materials. Besides, at the same temperature the result shows that the hydrogen storage capacity increases.

Based on the equilibrated models obtained from the GCMC calculations, MD simulations are carried out to investigate the diffusion of hydrogen inside CHC. Five pressure points (1, 5, 10, 15 and 20 bar) are selected along the adsorption isotherms of hydrogen at 77 K, 153 K, 193 K, 253 K and 298 K, respectively. MD simulations are performed for 100 ps in the NVT ensemble with a time step of 0.1 fs. The mean squared displacements (MSD) are calculated from the trajectories to study the diffusion of hydrogen atoms confined in the CHC. Supplementary Figure S1a–e shows the MSD-time curves in the logarithmic coordinates. On the log-log scale, the linear correlation between MSD and time is detected. The slopes of all the curves are around 1, showing an eventual diffusive behavior.

Figure 3b presents the variation of diffusion coefficients *D* of hydrogen adsorbed in CHC with pressure at 77 K, 153 K, 193 K, 253 K and 298 K. The resulting diffusion coefficients are in the range of 2.4 to 25.1 10^−4^*cm^2^/s, which is of the same order of magnitude as hydrogen diffusion in zeolites reported by Bar et al. experimentally [48]. At low pressure, the diffusion coefficient of hydrogen decreases rapidly with the increasing pressure, while the rate of decrease in the diffusion coefficient slows down at high pressure. Referring to Figure 3a,b, it can be seen that the variation of diffusion coefficients is related to the adsorption amount of hydrogen. The increases in pressure leads to an increase in the amount of adsorbed hydrogen, while the diffusion coefficient is reduced. At low pressure, as pressure increases the amount of adsorbed hydrogen increases dramatically, so the diffusion coefficient of hydrogen decreases rapidly. At the high-pressure zone, the diffusion coefficient drops slowly since hydrogen adsorption is close to saturation.

It can be seen from Figure 3b that the diffusion coefficient of adsorbed hydrogen at 298 K is higher than that at 77 K. At a low pressure of 1 bar, the diffusion coefficient for CHC increases by 3.2 times as temperature increases from 77 K to 298 K. The increase in temperature improves the intensity of the thermal motion of hydrogen molecules confined in microporous carbon, indicating that the diffusion of hydrogen molecules is a thermal activation process. Therefore, the diffusion coefficient increases as temperature increases.

## 4. Conclusions

In this study, the hydrogen adsorption capacity of the nanoporous materials of CHC is investigated by using grand canonical Monte Carlo simulations and molecular dynamics simulations. The result shows that low temperature and high pressure are conducive to hydrogen storage. At 77 K and 20 bars, the maximum amounts of adsorbed hydrogen (4.36 wt.% for CHC) are observed. In addition, based on the configuration obtained from GCMC simulations, the diffusion of hydrogen confined in the CHC is studied via MD simulations. Results show that the diffusion of hydrogen is both pressure- and temperature-dependent. The diffusion coefficient increases with a reduction in pressure since there is less hydrogen at low pressure. A high diffusion coefficient is computed at high temperature showing that hydrogen diffusion is a thermal activation process. In conclusion, as a newly nanoporous carbon, CHC with high stiffness could be a promising material for hydrogen storage.

It is worth noting that the classical MD simulations implemented in this study are unable to account for the quantum effect that might play roles at low temperatures close to zero. Keep in mind that hydrogen atoms follow one-dimensional motion along the nanopores of CHC. The average thermal de Broglie wavelength of such one-dimensional motion of hydrogen is 1.99, 1.41, 1.26, 1.1, and 1.0 Å at 77, 153, 193, 253, and 298 K, respectively, which is much less than the pore size of 8.3 Å. Therefore, the influence of the quantum effect will not be significant. In spite of the limitation of the method that ignores the quantum effect, our MD simulations have qualitatively captured the salient features of hydrogen behaviors in the nanopores of CHC.

## Supplementary Materials

The following are available online at https://www.mdpi.com/2079-4991/10/2/344/s1: Figure S1: Mean squared displacements for hydrogen confined in CHC under different pressures and temperature, Figure S2: Engineering stress-strain curves of carbon honeycomb for tensile loading, Video S1: the adsorption process of hydrogen at the pressure of 10 bar and the temperature of 77 K for CHC, Video S2: the stretching process along the z direction at the temperature of 77 K for CHC.
