# Supplementary material for: Adsorption and Diffusion of Hydrogen in Carbon Honeycomb
Source: Nanomaterials (Basel). 2020 Feb 18;10(2):344. doi: 10.3390/nano10020344 (PMC7075187; doi:10.3390/nano10020344)
Supplement: Supplementary file 1 [file nanomaterials-10-00344-s001.zip › supplementary/supplementry.docx]

**Supplementary Information**

Adsorption and diffusion of hydrogen in Carbon honeycomb

Qin Qin ^a^, Tingwei Sun ^a^, Hanxiao Wang ^b^, Haojie An ^a^, Lu Xie ^a,*^and Qing Peng ^c,*^

^a^ School of Mechanical Engineering, University of Science and Technology Beijing, Beijing 100083, PR China.

^b^ China Nuclear Power Technology Research Institute Co., Ltd., Reactor Engineering and Safety Research Center, Shenzhen 518031, China

^c^ Physics Department, King Fahd University of Petroleum & Minerals, Dhahran 31261, Saudi Arabia

*Corresponding authors: [xielu@ustb.edu.cn](mailto:xielu@ustb.edu.cn) (Lu Xie) and [qpeng.org@gmail.com](mailto:qpeng.org@gmail.com) (Qing Peng.)

**Supplementary Figures**


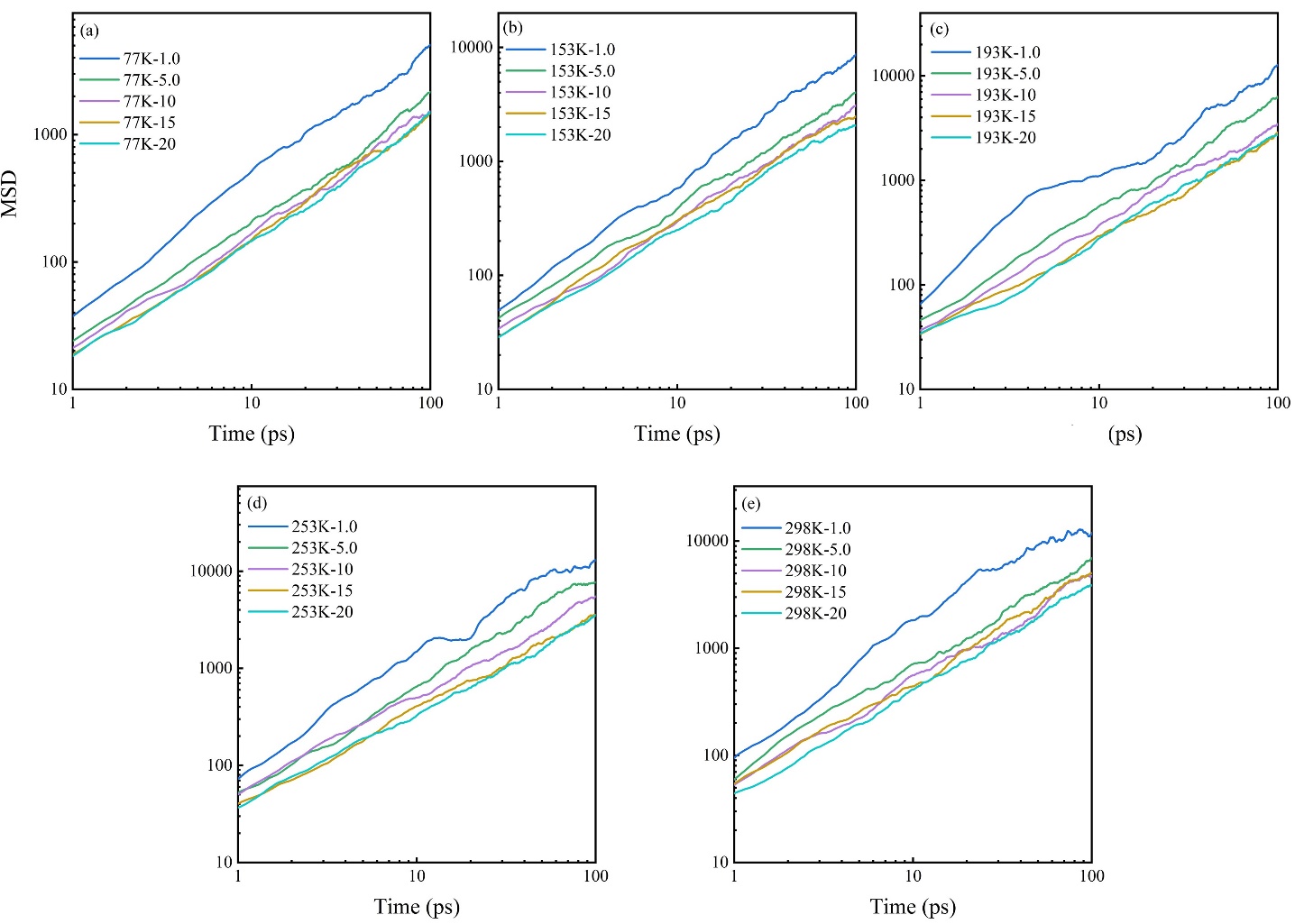


**Supplementary Figure S1.** Mean squared displacements for hydrogen confined in CHC under different pressures at the temperature of 77, 153 K, 193K, 253K and 298k, respectively.

In our previous research, it can be concluded that CHC has fascinating mechanical properties and potential application prospects [1, 2].The effect of temperature and vacancy-type defects on the mechanical properties of CHC was studied. We studied the mechanical properties of CHC when stretching along the different angles (zigzag, 4.7°, 16.1°, 19.1°, 25.3° and armchair). Defect 1 is a vacancy on the junction of CHC. Defect 2 and 3 are vacancy defects on the wall of CHC. Defect 2 is a kind of point vacancy near the junction. It can be found from Fig S2a that CHC has a yield stage in the cell axis direction since the break of C-C bonds on the junction. The tensile strength in the cell axial direction is higher than the zigzag direction and armchair direction at room temperature. CHC is an anisotropic material from the Fig S2a and b. Fig S2c shows that temperature affects the strength of the structure and the strength of CHC decreases with the temperature increasing. It can be found from Fig S2d that the strength is sensitive to the location and bonding of the vacancies in the cell axis (z) direction. It indicates that the absence of atoms at the junction has less effect on the structural strength.


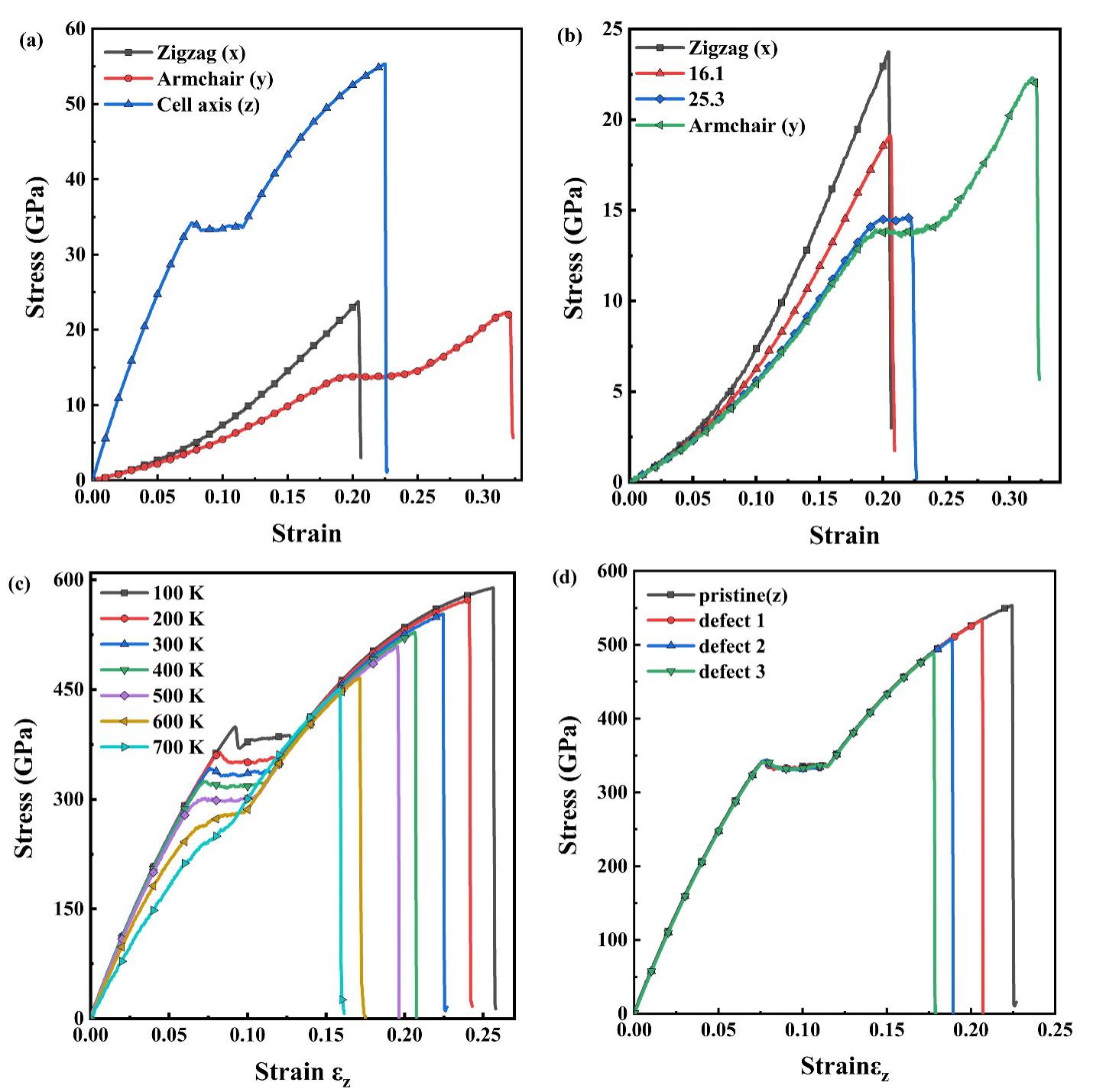


**Supplementary Fig. S2.** Engineering stress-strain curves of carbon honeycomb for tensile loading (a) along the zigzag (x), armchair (y) and cell axis (z) (b) stretching along the different angles at zigzag-armchair (x-y) plane (c) the temperature effect (d) the effect of vacancy-type defects in the cell axis (z) direction [1, 2].

**Supplementary Videos**

Supplementary Video S1 exhibit the adsorption process of hydrogen at the pressure of 10 bar and the temperature of 77 K for CHC. The amount of hydrogen adsorbed in the materials are also presented. Supplementary Video S2 exhibit the stretching process along the z direction at the temperature of 77 K for CHC

[1] L. Xie, H. An, C. He, Q. Qin, Q. Peng, Mechanical Properties of Vacancy Tuned Carbon Honeycomb, Nanomaterials, 9 (2019).

[2] Q. Qin, A. Haojie, H. Chenwei, X. Lu, P. Qing, Anisotropic and temperature dependent mechanical properties of carbon honeycomb, Nanotechnology, (2019).
